# Intrathecal, Not Systemic Inflammation Is Correlated With Multiple Sclerosis Severity, Especially in Progressive Multiple Sclerosis

**DOI:** 10.3389/fneur.2019.01232

**Published:** 2019-11-22

**Authors:** Joshua L. Milstein, Christopher R. Barbour, Kayla Jackson, Peter Kosa, Bibiana Bielekova

**Affiliations:** Neuroimmunological Diseases Section, National Institute of Allergy and Infectious Diseases (NIAID), National Institutes of Health (NIH), Bethesda, MD, United States

**Keywords:** multiple sclerosis, inflammation, systemic infections, cerebrospinal fluid, innate immunity, adaptive immunity, T cells, acute phase reactants

## Abstract

**Objective:** To test the hypothesis that Multiple Sclerosis (MS) patients have increased peripheral inflammation compared to healthy donors and that this systemic activation of the immune system, reflected by acute phase reactants (APRs) measured in the blood, contributes to intrathecal inflammation, which in turn contributes to the development of disability in MS.

**Methods:** Eight serum APRs measured in a prospectively-collected cross-sectional cohort with a total of 51 healthy donors and 291 untreated MS patients were standardized and assembled into related biomarker clusters to derive global measures of systemic inflammation. The resulting APR clusters were compared between diagnostic categories and correlated to equivalently-derived cerebrospinal fluid (CSF) biomarkers of innate and adaptive immunity. Finally, correlations were calculated between biomarkers of systemic and intrathecal inflammation and MS severity measures, which predict future rates of disability progression.

**Results:** While two blood APR clusters were elevated in MS patients, only one exhibited a weak correlation with MS severity. All CSF inflammation clusters, except CSF albumin, correlated with at least one measure of MS severity, with biomarkers of humoral adaptive immunity exhibiting the strongest correlations, especially in Progressive MS.

**Conclusion:** Systemic inflammation does not appear to be strongly associated with intrathecal inflammation in MS. Positive correlations between markers of intrathecal inflammation, especially of humoral immunity, with MS severity measures support a pathogenic role of intrathecal (compartmentalized) inflammation in central nervous system tissue destruction, including in Progressive MS.

## Introduction

Multiple Sclerosis (MS) is a chronic immune-mediated demyelinating disease of the central nervous system (CNS). Although the efficacy of immunomodulatory treatments declines as MS evolves (1), cellular and molecular cerebrospinal fluid (CSF) biomarkers have demonstrated a comparable amount of intrathecal inflammation in all three MS subtypes (2) [i.e., Relapsing-Remitting (RRMS), Primary Progressive (PPMS), and Secondary Progressive (SPMS)]. Furthermore, unbiased proteomic analysis of cerebrospinal fluid (CSF) biomarkers identified proteins that change with MS evolution (from early RRMS stage to late progressive MS stages), but none that could reproducibly differentiate PPMS and SPMS on a molecular level (3). These data are consistent with extensive genetic studies that also were unable to identify reproducible differences in MS susceptibility alleles between clinical subtypes of MS (4). The efficacy of B cell-depleting therapy (ocrelizumab) in both relapse-onset and PPMS clinical subgroups (5, 6) combined with clear and significant decline in efficacy on disability progression between young (<40 y) and older (≥40 y) relapse-onset MS patients (7), the genetic and proteomic results indicate that the MS disease process is largely overlapping, if not identical in all clinical MS subgroups and the main difference in the efficacy of current FDA-approved drugs on MS disability progression resides in the patient's age (1). The overlapping biology justifies merging PPMS and SPMS patients into a single Progressive MS (PMS) category, as is done in this study.

While it remains possible that inflammation, although present, no longer drives CNS tissue destruction in PMS, the partial efficacy of ocrelizumab in PPMS (5) and relapse-onset patients older than 40 years (7) makes the possibility of B cell-mediated intrathecal inflammation simply being an epiphenomenon in PMS unlikely. Additional support for the role of adaptive immunity in PMS comes from small clinical trials of natalizumab and methylprednisolone showing partial inhibition of the CSF T and memory B cell marker sCD27, which correlates with the inhibition of neurofilament light chain, a CSF marker of axonal dysfunction (8), and efficacy of siponimod in SPMS (9).

PMS patients often suffer from dysfunction of micturition, leading to urine retention and its bacterial colonization (10). Furthermore, clinical observations suggest that systemic infections may contribute to induction of relapses in RRMS patients (11). Therefore, we asked whether we could identify higher levels of biomarkers of systemic activation of immune responses reflected by acute phase reactants (APRs) in MS patients compared to healthy donors (HD) using a large Natural History cohort. While the phrase systemic inflammation is sometimes used as a broad term to point to a causal systemic autoimmune response, we use this term here as a means of indicating the presence of increased levels of blood APRs, as these are sensitive biomarkers of systemic infectious processes that activate innate and adaptive immunity. As it has been observed that some peripheral immune-mediated diseases can induce inflammation in the CNS (12), we additionally addressed the question that if MS patients do indeed have increased levels of peripheral APRs compared to HD, whether or not this systemic inflammation at least partially influences activation of innate and adaptive immunity in the CNS. Finally, we assessed the possible pathogenic role of systemic or intrathecal inflammation by correlating inflammatory biomarkers with MS severity, defined as speed of accumulation of MS disability.

## Materials and Methods

### Standard Protocol Approvals, Registrations, and Patient Consents

MS patients and healthy donor (HD) controls were selected from the National Institute of Allergy and Infectious Diseases (NIAID) Natural History cohort, prospectively collected between 6/2003 and 12/2017 under protocol “Comprehensive Multimodal Analysis of Neuroimmunological Diseases of the Central Nervous System” (registered at ClinicalTrials.gov under identifier: NCT00794352). The study was approved by the Central Neuroscience institutional review board of the National Institutes of Health (NIH) and all subjects signed informed consent.

Inclusion criteria for the patient population included: age of at least 12 years, presentation with a clinical syndrome consistent with immune-mediated CNS disorder or imaging evidence of inflammatory or demyelinating/dysmyelinating CNS disease, able to undergo required procedures, and able to provide informed consent on their own or via a Legally Authorized Representative or Durable Power of Attorney or parent/Legal Guardian in the case of minors. Inclusion criteria for healthy volunteers were comprised of the following conditions: at least 18 years old, no significant medical conditions, and normal vital signs at the time of screening. Patients and HDs underwent identical clinical, imaging, and research procedures. Research sample processing was performed blindly, using written standard operating procedures. The authors are able to provide informed consent upon request.

Diagnosis of MS was assigned after extensive diagnostic work-up and longitudinal follow-up as previously described (2). Data from MS patients who were on disease-modifying therapies (DMTs) within the 6 months prior to their visit (3 months for steroid administration for MS relapse) or had diagnoses of other inflammatory neurological disorders, non-inflammatory neurological disorders, or pediatric MS were excluded. All remaining eligible MS patients and HD with matched systemic and CSF inflammatory biomarkers were included in the analysis. Due to its exploratory nature, no power calculation was performed. In total, 51 HD and 291 patients with MS (1,163 total patient-visits) were used in the analyses.

Patient characteristics and demographics were compared between groups by using either Kruskal–Wallis tests, Mann–Whitney *U*-tests, or χ^2^-tests (Table 1). Patients with diagnoses of RRMS, PPMS, and SPMS were combined into a single MS group or patients with PPMS and SPMS were combined into a single PMS group and compared to the RRMS group for data analysis unless stated otherwise.

**Table 1:** Demographics and baseline measures for patients involved in the current study.

**Demographic variables**	**HD**	**RRMS**	**PMS**	***p-*value**
**Age (years)**				
Sample size	51	118	173	
Mean (SD)	39.4 (14.4)	42.3 (11.5)	55.3 (10.0)	<0.001
Range	19.4 – 71.3	18.0 – 76.4	22.0 – 70.8	
**Disease duration (years)**				
Sample size	NA	116	172	
Mean (SD)	NA	7.8 (7.8)	16.5 (10.3)	<0.001
Range	NA	0.03 – 42.8	0.9 – 48.4	
**Sex**				0.074
Sample size	51	118	173	
Male, *n* (%)	26 (51.0)	42 (35.6)	82 (47.4)	
Female, *n* (%)	25 (49.0)	76 (64.4)	91 (52.6)	
**Race**				<0.001
Sample size	36	117	169	
White, *n* (%)	15 (29.4)	81 (68.6)	139 (80.3)	
Black or African American, *n* (%)	15 (29.4)	30 (25.4)	23 (13.3)	
Other, *n* (%)	6 (11.8)	6 (5.1)	7 (4.1)	
**Family history of MS**				0.024
Sample size	29	82	144	
Yes, *n* (%)	2 (3.9)	27 (22.9)	40 (23.1)	
No, *n* (%)	27 (52.9)	55 (46.6)	104 (60.1)	
**History of smoking**				0.005
Sample size	35	84	143	
Yes, *n* (%)	22 (43.1)	53 (44.9)	116 (67.1)	
No, *n* (%)	13 (25.5)	31 (26.3)	27 (15.6)	
**MS-DSS**				<0.001
Sample size	46	108	167	
Mean (SD)	1.2 (0.1)	1.4 (0.6)	2.3 (1.1)	
Range	0.8 - 1.6	0.5 – 3.6	0.3 – 5.3	
**MSSS**				<0.001
Sample size	29	80	139	
Mean (SD)	1.8 (1.1)	4.2 (2.4)	6.7 (1.9)	
Range	0.9 – 4.31	0.2 – 9.27	0.72 – 9.97	
**ARMSS**				<0.001
Sample size	29	79	139	
Mean (SD)	1.2 (0.9)	3.8 (2.4)	6.3 (2.4)	
Range	0.2 – 3.8	0.5 – 9.4	0.8 – 9.9	

*Patient demographics and characteristics after excluding patients taking disease-modifying therapies and patients who were not healthy donors (HD) or who were diagnosed with a disease other than Relapsing-Remitting MS (RRMS) or Progressive MS (PMS). Differences in age and MS severity measures (MS-DSS, MSSS, ARMSS) were assessed using a Kruskal–Wallis test. A Mann-Whitney U-test was utilized for determining differences in disease duration between groups. All other patient demographic variables were analyzed via χ^2^ tests. Sample sizes and percentages for some demographic variables may not add up to the total group sample size or to 100% due to missing, unknown, or unreported data. Percentages for these variables are reported as a percentage of the total group sample size. ARMSS, Age Related Multiple Sclerosis Severity; HD, healthy donors; MS, Multiple Sclerosis; MS-DSS, Multiple Sclerosis-Disease Severity Scale: MSSS, Multiple Sclerosis Severity Score; RRMS, Relapsing-Remitting Multiple Sclerosis; PMS, Progressive Multiple Sclerosis; SD, standard deviation*.

### Data Collection and Measurement

Blood samples and small aliquots of CSF were processed by the NIH Clinical Center laboratory. CSF albumin (mg/dL), IgG levels (mg/dL), IgG index and the serum inflammatory mediators (acute phase reactants; APRs) albumin (g/dL), ceruloplasmin (mg/dL), white blood cell count (WBC; cells/μL), C-reactive protein (CRP; mg/L), erythrocyte sedimentation rate (ESR), iron (mcg/dL), ferritin (mcg/L), and transferrin (mg/dL) were reported in patients' electronic medical records. CSF samples were collected and processed by the Neuroimmunological Diseases Section (NDS) using a published standardized protocol (2) where the investigators were blinded as to the diagnoses of the patients. CSF markers of myeloid lineage cells sCD14 (ng/mL), sCD163 (ng/mL), and chitinase-3-like 1 (CHI3L1; ng/mL) as well as soluble B cell maturation antigen (sBCMA; pg/mL) and the T and memory B cell activation biomarker sCD27 (U/mL) were assessed via optimized electrochemiluminescence-based ELISAs using the Meso Scale Discovery® platform (MSD; Meso Scale Diagnostics, Rockville, MD, https://www.mesoscale.com/) as described (2).

The MS severity was measured by MS Severity Score (MSSS; 12), Age-Related MS Severity Score [ARMSS; (13)], and MS-Disease Severity Scale (MS-DSS) a new, statistical-learning-derived scale capable of predicting future rates of disability accumulation (14). The algorithm for MS-DSS calculation is publicly available at https://bielekovalab.shinyapps.io/msdss/.

### Statistical Analyses

Intraclass correlation coefficients (ICCs) were created for each marker in order to examine the stability of measurements within individual patients. As most markers (except serum albumin, WBC, and iron) had ICCs > 0.8, indicating stability of biomarker measurements within patients, all data available for each biomarker across all distinct visits per patient were averaged to derive more “stable” measures of inflammation. For MS severity, only the most recent MSSS, ARMSS, and MS-DSS scores were used for each patient, due to previously-validated high correlations with longitudinally-measured disability progression slopes (14). The resulting biomarkers and severity scale data were matched, transformed using Box-Cox transformations, and subsequently converted into standardized Z scores.

Differences in biomarkers between healthy donors and MS patients were examined using analysis-of-variance from a linear regression model, where confounding variables on biomarker values, including patient race, age, sex, and the interaction between them on individual APRs were included if effects were present. In order to correct for the effects of confounding factors on individual APRs, the estimated model effects (excluding diagnosis) were subtracted from the marker measurements. These residuals after adjusting biomarker Z scores for race, age, and sex were used for subsequent analyses, as they represent the proportion of variance that is attributable to the APR, and not to race, age, and sex covariates.

To develop more global measures of inflammation, blood APRs whose Z score residuals correlated with each other according to their Pearson correlation coefficients were classified as a single biomarker cluster. All patients were examined when defining blood clusters, while only MS patients were considered when constructing CSF clusters, as only MS patients had intrathecal inflammation. In order to create these clusters, the residuals from each biomarker comprising a defined cluster were averaged per patient, resulting in a single cluster score per patient. For clustering purposes and for ease of interpretation, the Z scores of the negative APRs serum albumin, iron, and transferrin were multiplied by −1 after being adjusted for age and sex since levels of these APRs decrease during inflammation. The resulting “inverted” Z score residuals were correlated with the positive blood APRs and biomarkers with non-missing values were subsequently averaged into their respective biomarker clusters. While all CSF biomarker residuals utilized for this study were found to be highly correlated, to gain insight as to which aspects of neuroinflammation are affected by blood APRs, the CSF markers of activated myeloid cells sCD14, sCD163, and CHI3L1 were grouped into one cluster representing activation of innate immunity while CSF IgG levels, IgG index, and sBCMA were grouped into a single cluster representing humoral immunity. Levels of CSF albumin and sCD27, a biomarker that is released predominantly by activated T cells (although memory B cells and plasma cells may also release sCD27 at low levels), were analyzed individually due to their distinct biological implications. Pearson correlations were calculated between blood and CSF biomarker cluster scores and then again between these cluster scores and MS severity measures for all MS patients in addition to the RRMS and PMS sub-cohorts. As the MS severity measures exhibited right-skew, Spearman correlation coefficients were used to examine relationships between cluster scores and MS severity measures. The *p*-values for these correlations and for comparisons between diagnosis were adjusted to account for multiple comparisons using the False Discovery Rate (FDR) method and these adjusted *p*-values are those reported in the figures and manuscript unless otherwise stated (15).

The threshold for statistical significance for the present study was set at *p* < 0.05 for all comparisons. Certain biomarker and clinical data were not available for all patients/all data-points due to scheduling conflicts or technical problems. Pairwise complete observations were used when examining potential relationships. The sample sizes for various comparisons are provided throughout the tables and figures. All statistical analyses were conducted using R (16), and GraphPad Prism 7 (GraphPad Software, La Jolla, CA).

## Results

### MS Patients Have Higher Levels of Some Blood APRs After Adjusting for Race, Age, and Sex

**Figure 1:**
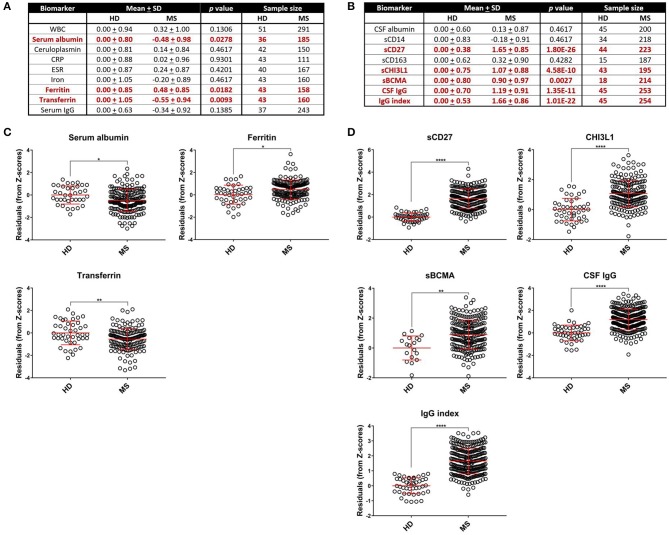
Comparing HD and MS biomarker residual levels after adjusting for race, age, and sex. Mean comparisons between HD and MS biomarker Z score residuals after matching and adjusting for race, age, and sex and *p-*values after adjusting for multiple comparisons are shown. All comparisons are shown in table format in **(A,B)** and graphical depictions are also presented for blood APRs **(C)** and CSF markers **(D)** individually that demonstrated differences between HD and MS patients. MS patients had higher levels of ferritin in the blood and elevated levels of CHI3L1, sCD27, sBCMA, IgG, and IgG index in the CSF. Serum albumin and transferrin were decreased in MS patients, which is suggestive of an increased inflammatory profile due to these proteins being negative APRs. *p < 0.05, **p < 0.01, ****p < 0.0001. APR, acute phase reactant; CHI3L1, chitinase-3-like 1; CRP, C-reactive protein; CSF, cerebrospinal fluid; ESR, erythrocyte sedimentation rate; HD, healthy donor; IgG, immunoglobulin G; MS, Multiple Sclerosis; sBCMA, soluble B cell maturation antigen; sCD, soluble cluster of differentiation; SD, standard deviation; WBC, white blood cell count.

To limit the extraneous variables that may influence our results, we employed a linear regression model to adjust for race, age, and sex for biomarkers where these variables were found to influence biomarker level (see section Materials and Methods for details). We refer to these covariate-adjusted values of measured biomarkers as Z score residuals. After accounting for these effects (if present), we found that MS patients had an elevated amount of ferritin (Figures 1A,C; *p* = 0.0182) and CSF CHI3L1, sBCMA, CSF IgG, IgG index, and sCD27 (Figures 1B,D; *p* = 0.0028 for sBCMA and *p* < 0.0001 for all other markers) as well as diminished levels of the negative blood APRs serum albumin and transferrin (Figure 1C; *p* = 0.0278 and *p* = 0.0093, respectively). We also assessed the differences of adjusted blood and CSF biomarker Z score residuals between HD, RRMS, and PMS and found that all of these biomarkers were also significantly different between HD and both MS groups, with the exception of serum albumin, which was only decreased in PMS patients (Supplementary Figure 1). Importantly, only iron and sBCMA were significantly different between PPMS and SPMS groups (Supplementary Figure 2; *p* = 0.0498 and *p* = 0.0111, respectively).

**Figure 2:**
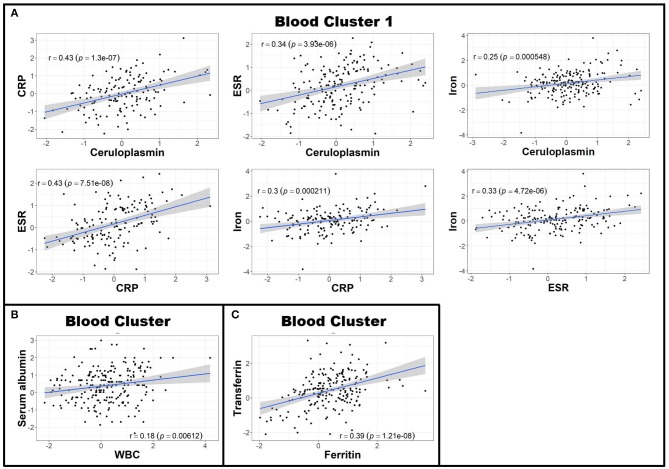
Blood biomarkers can be clustered to create more globalized, biologically-relevant measures of systemic inflammation. Z scores of negative APRs in the blood (serum albumin, iron, and transferrin) were first multiplied by −1 and subsequently adjusted for race, age, and sex. Afterwards, Pearson correlations between individual blood biomarker Z score residuals were calculated. Biomarkers that correlated were subsequently grouped into clusters. This resulted in three separate clusters: the first containing ceruloplasmin, CRP, ESR, and iron **(A)**; the second comprised of serum albumin and WBC **(B)**; and the third with ferritin and transferrin **(C)**. The axes are selected to have better visual assessment of majority of patients' biomarkers. Thus, a few individual points may be missing in these graphs. APR, acute phase reactant; CRP, C-reactive protein; ESR, erythrocyte sedimentation rate; MS, Multiple Sclerosis; WBC, white blood cell count.

**Figure 3:**
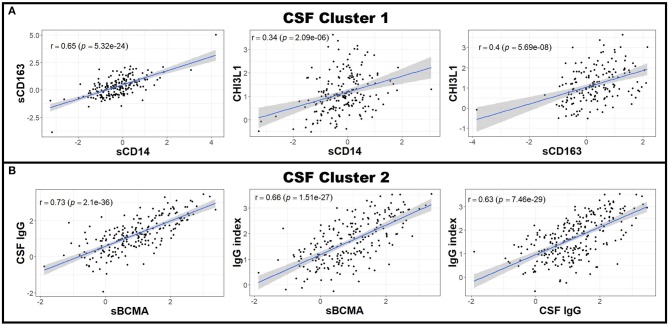
CSF biomarkers can be clustered into biologically-relevant measures of intrathecal inflammation. Pearson correlations between individual CSF biomarker Z score residuals were calculated. Biomarkers that correlated with others were grouped into clusters. Because nearly all of the measured CSF biomarkers correlated with each other in patients with MS (data not shown), two clusters were made; the first using the biologically-related CSF myeloid lineage markers CHI3L1, sCD14, and sCD163 **(A)** and the second comprising of sBCMA, CSF IgG, and IgG index **(B)**. All other CSF biomarkers not included in these clusters (CSF albumin and sCD27) were used for future analyses as standalone proteins. The axes are selected to have better visual assessment of majority of patients' biomarkers. Thus, a few individual points may be missing in these graphs. CHI3L1, chitinase-3-like 1; CSF, cerebrospinal fluid; IgG, immunoglobulin G; MS, Multiple Sclerosis; sBCMA, soluble B cell maturation antigen; sCD, soluble cluster of differentiation.

Next, to reduce dimensionality and strengthen the robustness of analyses, we derived more global measures of systemic inflammation, as was done previously for Alzheimer's Disease (17). To do so, we first multiplied the Z scores of the negative blood APRs serum albumin, iron, and transferrin by −1 (since the levels of these proteins decrease in response to inflammation) after adjusting for confounding factors as described in the Methods. Pearson correlations between pairs of biomarker Z score residuals were then calculated to create biologically-relevant biomarker clusters. These clusters were dichotomized between blood and intrathecal inflammatory biomarkers so that blood biomarkers were only clustered with other blood biomarkers and vice versa with CSF biomarkers. This method identified three separate biomarker clusters in the blood (Figure 2). Blood cluster 1 contained ceruloplasmin, C-reactive protein (CRP), erythrocyte sedimentation rate (ESR), and iron. Blood cluster 2 consisted of white blood cell count (WBC) and serum albumin, while ferritin and transferrin comprised Blood cluster 3. Although in the CSF we found that, aside from CSF albumin, all CSF biomarkers correlated with each other, we kept inflammatory biomarkers of activated myeloid lineage cells (sCD14, sCD163, and CHI3L1; CSF cluster 1) separate from biomarkers of humoral immunity (IgG, IgG index, and sBCMA; CSF cluster 2) and from sCD27 (a biomarker predominantly secreted by activated T cells, but also released in lower quantities by other immune cell types; (2) and CSF albumin (Figure 3). This separation allowed us to investigate the effects of systemic inflammation on different components of intrathecal immunity separately. Correlations between blood and CSF biomarker clusters and the biomarkers that comprise them are shown graphically in Supplementary Figure 3.

**Figure 4:**
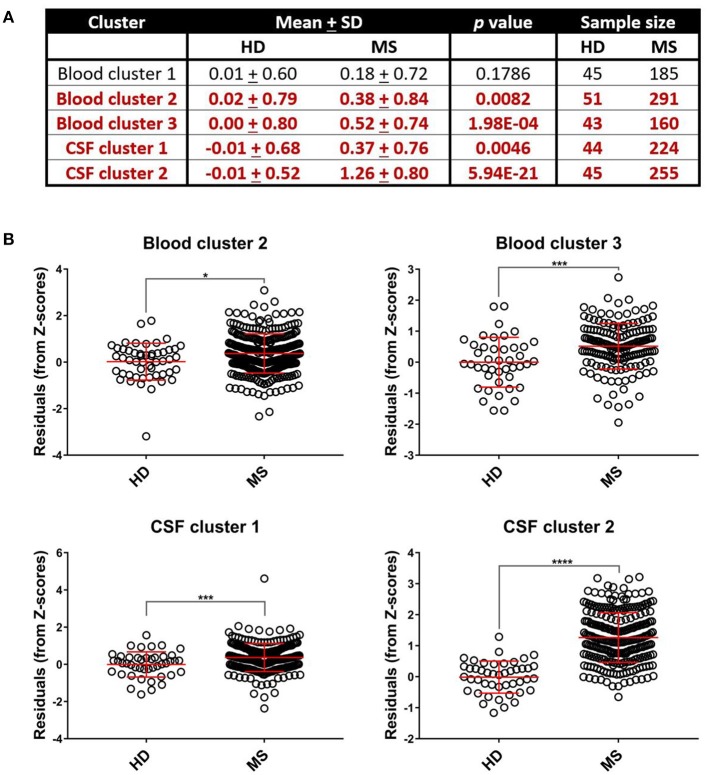
Select systemic and CSF inflammatory clusters are upregulated in MS patients. Differences between mean biomarker cluster scores between HD and MS groups after adjusting for multiple comparisons were assessed using unpaired *t*-tests **(A)**. We found that MS patients had elevated cluster scores compared to healthy donors for Blood cluster 2, Blood cluster 3, CSF cluster 1, and CSF cluster 2 **(B)**. *p < 0.05, ***p < 0.001, ****p < 0.0001. CSF, cerebrospinal fluid; HD, healthy donor; MS, Multiple Sclerosis; SD, standard deviation.

While Blood cluster 1 was not elevated in MS patients compared to HD (Figure 4; *p* = 0.1786), both Blood cluster 2 and Blood cluster 3 were increased in MS (Figure 4; *p* = 0.0082 and *p* = 0.0002, respectively). MS patients also exhibited higher cluster scores for CSF cluster 1 and CSF cluster 2 compared to HD (Figure 4; *p* = 0.0046 and *p* < 0.0001, respectively). Notably, sCD27 correlated strongly with both CSF cluster 1 and CSF cluster 2 (Supplementary Figure 5), although, intuitively, this correlation was stronger with CSF cluster 2 than CSF cluster 1. This indicates that while activation of innate immunity, represented by myeloid lineage markers, correlates with activation of adaptive immunity in MS, these correlations are stronger between two arms of adaptive immunity: the humoral arm, represented predominantly by CSF cluster 2, and the T cell arm, represented by sCD27. When the MS cohort was split into RRMS and PMS groups, the PMS group showed higher levels of all blood biomarker clusters (*p* = 0.0322, *p* = 0.0080, and *p* = 0.0088 for Blood clusters 1, 2, and 3, respectively) and the RRMS group had increased amounts of Blood cluster 3 compared to HD (Supplementary Figure 4; *p* < 0.0001). Additionally, we found that, compared to controls, PMS patients had elevated levels of CSF cluster 1, CSF cluster 2, and sCD27 (Supplementary Figure 4; *p* = 0.0202 for CSF cluster 1 and *p* < 0.0001 for both CSF cluster 2 and sCD27), whereas RRMS patients showed increased amounts of CSF cluster 2 and sCD27 compared to HD (Supplementary Figure 4; *p* < 0.0001 for both clusters).

### Blood APR Clusters Do Not Positively Correlate With CSF Biomarkers of Innate and Adaptive Immunity

Having established more global, biologically-relevant measures to assess the inflammatory status of MS patients compared to healthy donors, we aimed to address the hypothesis that systemic APRs contribute to the proinflammatory environment in the CNS of MS patients. For this, correlations between biomarker clusters from the blood and CSF were calculated in the MS cohort. We did not observe a positive correlation between blood inflammatory clusters and any of the biomarkers of CSF inflammation (Table 2). However, there was a trend toward a positive correlation between Blood cluster 1 (ceruloplasmin, CRP, ESR, and iron) and CSF cluster 2 (sBCMA, CSF IgG, and CSF IgG Index; *r* = 0.24, *p* = 0.053) in the RRMS cohort only (Table 3).

**Table 2:** Inflammatory blood biomarker clusters do not positively correlate with CSF inflammation in MS patients.

**Variable 1**	**Variable 2**	**Pearson r**	***p*-value**	***N***
Blood cluster 1	CSF cluster 1	−0.11	0.713	153
Blood cluster 1	CSF cluster 2	0.07	0.751	164
Blood cluster 1	sCD27	−0.01	0.989	153
Blood cluster 1	CSF albumin	0	0.989	135
Blood cluster 2	CSF cluster 1	0.05	0.751	224
Blood cluster 2	CSF cluster 2	0.13	0.411	255
Blood cluster 2	sCD27	0.09	0.713	223
Blood cluster 2	CSF albumin	0.08	0.751	200
Blood cluster 3	CSF cluster 1	0.07	0.751	134
Blood cluster 3	CSF cluster 2	0.03	0.9	147
Blood cluster 3	sCD27	0	0.989	134
Blood cluster 3	CSF albumin	−0.06	0.751	123

*Pearson correlations between blood and CSF clusters were conducted using all MS patients to determine whether blood APRs were associated with CSF inflammation and neurodegeneration. All correlation coefficients, p values after adjusting for multiple comparisons, and sample sizes are shown. APR, acute phase reactant; CSF, cerebrospinal fluid; MS, Multiple Sclerosis; sCD, soluble cluster of differentiation*.

**Table 3:** Inflammatory blood biomarker clusters do not positively correlate with CSF inflammation and neurodegeneration in RRMS or PMS patients.

**RRMS**					**PMS**				
**Variable 1**	**Variable 2**	**Pearson r**	***p*-value**	***N***	**Variable 1**	**Variable 2**	**Pearson r**	***p*-value**	***N***
Blood cluster 1	CSF cluster 1	−0.05	0.878	257	Blood cluster 1	CSF cluster 1	0.05	0.981	87
Blood cluster 1	CSF cluster 2	0.24	0.053	292	Blood cluster 1	CSF cluster 2	0.06	0.981	90
Blood cluster 1	sCD27	0.18	0.185	256	Blood cluster 1	sCD27	0.01	0.981	87
Blood cluster 1	CSF albumin	−0.05	0.878	238	Blood cluster 1	CSF albumin	0.09	0.981	76
Blood cluster 2	CSF cluster 1	0.01	0.982	268	Blood cluster 2	CSF cluster 1	0	0.981	130
Blood cluster 2	CSF cluster 2	0.07	0.878	300	Blood cluster 2	CSF cluster 2	0.07	0.981	150
Blood cluster 2	sCD27	0.03	0.969	267	Blood cluster 2	sCD27	0.03	0.981	129
Blood cluster 2	CSF albumin	0.15	0.288	245	Blood cluster 2	CSF albumin	0.14	0.826	120
Blood cluster 3	CSF cluster 1	−0.03	0.969	173	Blood cluster 3	CSF cluster 1	0.01	0.981	83
Blood cluster 3	CSF cluster 2	0.06	0.878	187	Blood cluster 3	CSF cluster 2	0.05	0.981	88
Blood cluster 3	sCD27	0	0.982	173	Blood cluster 3	sCD27	−0.02	0.981	83
Blood cluster 3	CSF albumin	−0.24	0.185	163	Blood cluster 3	CSF albumin	−0.22	0.658	74

*Pearson correlations between blood and CSF clusters were conducted using RRMS or PMS patient data to determine whether blood APRs were associated with CSF inflammation. All correlation coefficients, p-values after adjusting for multiple comparisons, and sample sizes are shown. APR, acute phase reactant; CSF, cerebrospinal fluid; PMS, Progressive Multiple Sclerosis; RRMS, Relapsing-Remitting Multiple Sclerosis; sCD, soluble cluster of differentiation*.

### CSF Markers of Innate and Adaptive Immunity Correlate With MS Severity

**Figure 5:**
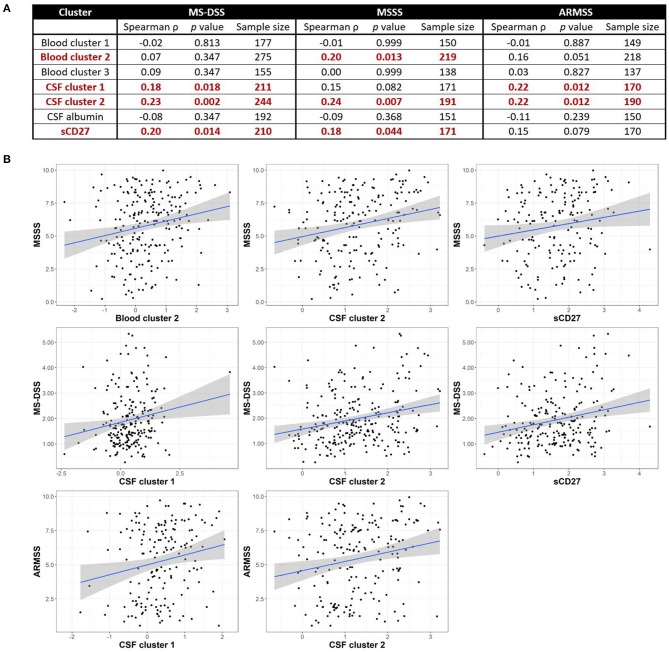
Inflammatory blood and CSF immune biomarkers correlate with MS severity among all MS patients combined. Spearman correlations between all blood and CSF clusters and MS-DSS, MSSS, and ARMSS using all MS patients are shown after adjusting for multiple comparisons **(A)**. Relationships between cluster scores and severity measures with *p* < 0.05 are bolded and graphically depicted **(B)**. The axes are selected to have better visual assessment of majority of patients' cluster scores. Thus, a few individual points may be missing in these graphs. ARMSS, Age Related Multiple Sclerosis Severity; CSF, cerebrospinal fluid; MS, Multiple Sclerosis; MS-DSS, Multiple Sclerosis-Disease Severity Scale; MSSS, Multiple Sclerosis Severity Score.

**Figure 6:**
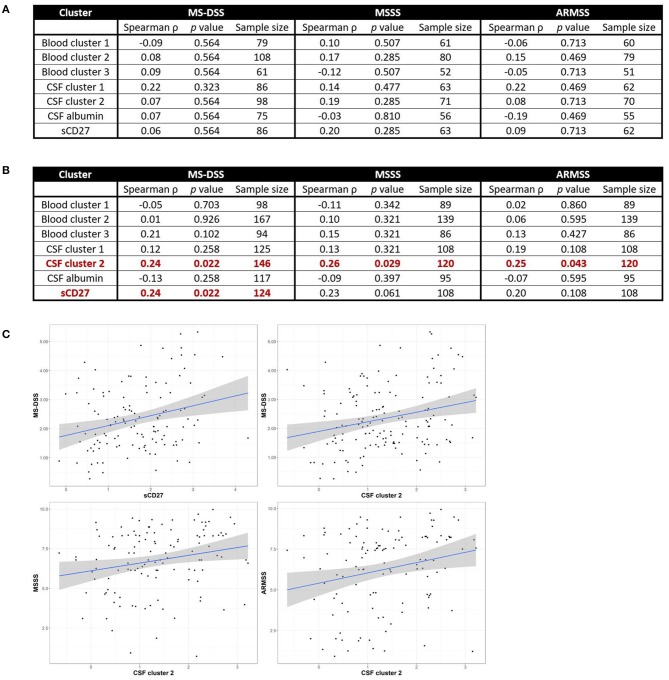
CSF biomarkers of innate and adaptive immunity correlate with MS severity in subjects with PMS, but not RRMS. Spearman correlations between all blood and CSF clusters and MS-DSS, MSSS, and ARMSS in only RRMS **(A)** or Progressive MS **(B)** patients are shown after adjusting for multiple comparisons. Relationships between cluster scores and severity measures with *p* < 0.05 are bolded and graphically depicted **(C)**. The axes are selected to have better visual assessment of majority of patients' cluster scores. Thus, a few individual points may be missing in these graphs. ARMSS, Age Related Multiple Sclerosis Severity; CSF, cerebrospinal fluid; MS, Multiple Sclerosis; MS-DSS, Multiple Sclerosis-Disease Severity Scale; MSSS, Multiple Sclerosis Severity Score.

Although we observed no positive correlations between biomarkers of systemic and intrathecal inflammation, we asked whether systemic inflammation may contribute to MS severity, defined as the rate of disability progression, by mechanisms other than driving intrathecal activation of innate or adaptive immunity. In a similar way, we also addressed the hypothesis that different types of intrathecal inflammation contribute to CNS damage in MS by correlating CSF inflammatory biomarkers with MS severity. Thus, we evaluated correlations between blood and CSF inflammatory biomarker clusters and three measures of MS severity [MSSS, ARMSSS, and MS-DSS; (18)] in the entire MS cohort (Figure 5) and the RRMS and PMS sub-cohorts (Figure 6).

For clusters of systemic inflammation, we observed a positive correlation between blood cluster 2 (WBC and serum albumin) with MSSS (ρ = 0.20*, p* = 0.013) in all MS patients (Figure 5). We observed no correlations between clusters of systemic inflammation and any measure of MS severity in RRMS or PMS patients (Figure 6). In contrast, after adjusting for multiple comparisons, both CSF cluster 2 and sCD27 correlated with at least one measure of MS severity in all MS patients (Figure 5) as well as PMS patients individually (Figure 6). The innate myeloid lineage biomarker cluster (sCD14, sCD163, and CHI3L1; CSF cluster 1) correlated with ARMSS (Figure 5; ρ = 0.22, *p* = 0.012) and the T and memory B cell marker sCD27 correlated with both MS-DSS (ρ = 0.20, *p* = 0.014) and MSSS (ρ = 0.18, *p* = 0.044) in the whole MS cohort (Figure 5). Surprisingly, the correlation between sCD27 and MS-DSS was stronger after analyzing only the PMS sub-cohort (Figure 6; ρ = 0.24, *p* = 0.022). Finally, the humoral immunity cluster (CSF IgG, IgG index, and sBCMA; CSF cluster 2) correlated with MS severity in the whole MS cohort (Figure 5; ARMSS ρ = 0.22, *p* = 0.012; MSSS ρ = 0.24, *p* = 0.007; and MS-DSS ρ = 0.23, *p* = 0.002) as well as in the PMS sub-cohort (Figure 6; ARMSS ρ = 0.25, *p* = 0.043; MSSS ρ = 0.26, *p* = 0.029; and MS-DSS ρ = 0.24, *p* = 0.022).

## Discussion

Infections may play a role in initiating relapses and activating innate and adaptive immune cells in individuals with RRMS (11, 19). They may also play an essential role in MS etiology [especially Epstein-Barr Virus Infection (20, 21)]. Most of these studies have focused on the induction of autoreactive T cells (22), but the connection between the immunological responses to systemic infections and MS pathology has proven to be more elusive. The current study assessed whether MS patients had higher levels of systemic inflammation compared to HD and if this influenced intrathecal inflammation and MS disease severity. We found that MS patients had significantly higher levels of some inflammatory blood biomarkers, but we were unable to demonstrate a strong association between biomarkers of systemic inflammation and intrathecal inflammation or MS severity. Because we did not take into account the infection status of the patients used for these analyses or whether the patients diagnosed with RRMS were in remission or experiencing a relapse, our findings are not necessarily contradictory to those suggesting a role for systemic infection in inducing relapses in these RRMS patients (11, 21). Assessing this in future studies could elaborate more on the effect of systemic infection on induction of MS relapses.

While we observed that MS patients have higher levels of systemic inflammation than healthy donors, this was true only for Blood cluster 2 (WBC and serum albumin) and Blood cluster 3 (ferritin and transferrin; Figure 4). Nevertheless, none of the blood inflammation clusters correlated positively with any of the inflammatory CSF clusters in either all MS patients combined or in the separated RRMS and PMS groups. When assessing correlations between systemic inflammation and MS severity, Blood cluster 2 exhibited a marginal correlation with MS severity in the whole MS cohort (Figure 5) in one of the severity measures used, but not in RRMS or PMS cohorts alone (Figure 6). The low proportion of variance of MS severity explained by Blood cluster 2 and the lack of correlation in phenotypically homogeneous MS subgroups suggest that the observed effect on MS severity is either attributable to Type 1 statistical error or the influence of systemic inflammation on MS severity occurs in both MS subtypes, but it is so small that dividing the MS cohort on RRMS and PMS left both underpowered to detect this small effect size. The lack of correlation between systemic inflammatory biomarkers and MS severity in PMS rejects the hypothesis that systemic inflammation that is typically associated with urinary tract infections and release of pathogen-associated molecular patterns into circulation represents an important driver of intrathecal inflammation and disability in subjects with PMS.

In contrast, we observed an association between intrathecal inflammation and multiple MS severity outcomes, even though the proportion of variance explained was small. The positive correlations between intrathecal inflammatory biomarkers and MS severity was stronger for PMS patients, as compared to the whole MS cohort (Figure 5) and the RRMS sub-cohort (Figure 6). This is likely due to the fact that disability progression in RRMS may occur below the clinical detection threshold, or is masked by repair mechanisms, such as remyelination and development of new synaptic circuits. Nevertheless, the stronger correlations in the PMS cohort extend our previous inference about the relevance of intrathecal inflammation in the late stages of MS. In previous CSF studies, we found that the amount of intrathecal inflammation is comparable between RRMS and the two Progressive MS subtypes, but that in PMS, the intrathecal inflammation is compartmentalized to CNS tissue (2, 3), and adaptive immune cells are terminally-differentiated (23). These two characteristics likely underlie low/absent efficacy of current immunomodulatory DMTs in subjects with PMS over the age of 55 (1). This paper extends these observations, as our findings suggest that ongoing intrathecal inflammation in PMS is pathogenic and continues influencing CNS tissue destruction and therefore, MS severity, although we must stress that the percentage of variance explained by intrathecal inflammation is low. While correlations do not necessarily imply causality, the efficacy of next generation DMTs such as ocrelizumab (5) and siponimod (9) in younger subjects with PMS supports this conclusion. The strength of correlations with MS severity measures suggests that adaptive immunity, especially its humoral aspect (B cells, plasma cells, plasmablasts, and possibly antibodies), may contribute to CNS tissue destruction more than innate immunity, as represented here by biomarkers of myeloid lineage cells. However, this conclusion is only tentative because the differences in the strength of correlations are small and the studied biomarkers do not comprise all phenotypes of myeloid cells. Therefore, use of different biomarkers in future studies may alter this conclusion. Nevertheless, the possible pathogenic role of B cells in MS is supported by the high comparative age-adjusted efficacy of B cell-depleting treatments, such as ocrelizumab and rituximab, in MS (1) in comparison to DMTs with different mechanisms of action. Possible mechanisms of CNS tissue destruction by humoral immunity (24, 25) have highlighted antigen-presentation and cytokine secretion. An unbiased assessment of CSF B cells emphasized their pro-lymphangiogenic potential, which may play an important role in the compartmentalization of MS inflammation to CNS tissue (26). In addition, other reports have shown increased sBCMA in the CSF of MS patients (27).

A limitation of the current study is its exploratory, retrospective analyses of the prospectively acquired data, lacking a pre-determined power analysis, which could have led to an underpowered study. Arguably, if this study failed to detect true positive relationships between systemic and intrathecal inflammation and between systemic inflammation and MS severity, then such relationships would likely be too small to be clinically-relevant. Conversely, the positive relationships identified in this study between intrathecal inflammation and MS severity should be independently validated, in a properly-powered, new MS cohort before they can be accepted as generalizable. Furthermore, the current study did not measure all biomarkers that have been reported as elevated in MS. Instead, based on previous measurements of CSF biomarkers throughout the course of the Natural History protocol (2), we selected for analysis in the current study only those that demonstrated the greatest differences between MS and healthy volunteers and could be attributed to either innate or adaptive immunity. In contrast, the blood biomarkers were part of a standard inflammation panel to assess the possible presence of a systemic infection. We acknowledge that a variety of other biomarkers have been shown to be elevated in the serum of MS patients, including the adhesion molecule sICAM-1 (28), the matrix metalloproteinase MMP9 (29), and glial fibrillary acidic protein (30). Similarly, we acknowledge that, while sCD27 is produced in highest per cell quantities by activated CD8^+^ compared to CD4^+^ T cells (2), many other cells express sCD27 either as mRNA or as a cell surface marker. For example, a population of antigen-primed CD27^+^CD70^+^ memory B cells has been identified in secondary lymphoid tissue (31) and CD27^hi^CD38^hi^IgD^−^ B cells have been observed bi-compartmentally in the blood and CSF of MS patients (32). As we found that sCD27 correlated with both CSF cluster 1 and CSF cluster 2 (Supplementary Figure 5), how much these sub-populations of sCD27-expressing B cells or other cell types contributed to the sCD27 levels measured in the current study is not known. While we combined PPMS and SPMS groups into a single PMS group for the majority of our analyses based on the rationale explained in the introduction, we do not rule out the possibility that there may be subtle biological differences between PPMS and SPMS patients that may be identified in larger studies. Indeed, we observed differences in blood iron levels and CSF sBCMA (Supplementary Figure 2). To our knowledge, even though biological differences between PPMS and SPMS patients may have been suggested in some previous publications, the level of evidence (reflected by study design that limits bias, strength of *p*-values after adjustments for multiple comparisons and lack of replication in an independent cohort) that such differences are generalizable, is currently lacking. A final limitation for the current study is that it does not address mechanisms underlying the correlations between the biomarkers used and MS severity.

The correlation between CSF inflammatory biomarkers and the rate of disability progression in MS in general and particularly in PMS validates ongoing intrathecal inflammation, especially its humoral arm, as an important therapeutic target in MS. It is likely that effective inhibition of intrathecal inflammation in PMS and residual inflammation in RRMS patients on current DMTs will require a new generation of CNS-penetrant drugs that inhibit effector immune responses outside of the proliferation cycle.

## Data Availability Statement

All datasets generated for this study are included in the article/Supplementary Material.

## Ethics Statement

The studies involving human participants were reviewed and approved by Central Neuroscience institutional review board of the National Institutes of Health (NIH). The patients/participants provided their written informed consent to participate in this study.

## Author Contributions

JM analyzed and interpreted the data and drafted the manuscript for intellectual content. CB provided guidance on the data analysis and interpretation of the data as well as revised the manuscript for intellectual content. KJ assisted in analysis of the data and revised the manuscript for intellectual content. PK assisted in the collection and maintenance of the data and revised the manuscript for intellectual content. BB designed and conceptualized the study and was involved in interpretation of the data and drafting of the manuscript for intellectual content.

### Conflict of Interest

The authors declare that the research was conducted in the absence of any commercial or financial relationships that could be construed as a potential conflict of interest.

## Abbreviations

APR: acute phase reactant
CHI3L1: chitinase-3-like 1
CNS: central nervous system
CRP: C-reactive protein
CSF: cerebrospinal fluid
ESR: erythrocyte sedimentation rate
IgG: immunoglobulin G
MS: Multiple Sclerosis
MS-DSS: Multiple Sclerosis-Disease Severity Scale
PMS: Progressive MS
PPMS: Primary Progressive MS
RRMS: Relapsing-Remitting MS
sBCMA: soluble B cell maturation antigen
sCD: soluble cluster of differentiation
SPMS: Secondary Progressive MS
WBC: white blood cell count. ARMSS, Age-Related Multiple Sclerosis Severity Score
FDR: False Discovery Rate
ICC: interclass correlation
MS-DSS: Multiple Sclerosis Diseases Severity Scale
MSSS: Multiple Sclerosis Severity Score
SD: standard deviation.
