# Supplementary figures and images for: Intrathecal, Not Systemic Inflammation Is Correlated With Multiple Sclerosis Severity, Especially in Progressive Multiple Sclerosis
Source: Front Neurol. 2019 Nov 22;10:1232. doi: 10.3389/fneur.2019.01232 (PMC6884093; doi:10.3389/fneur.2019.01232)

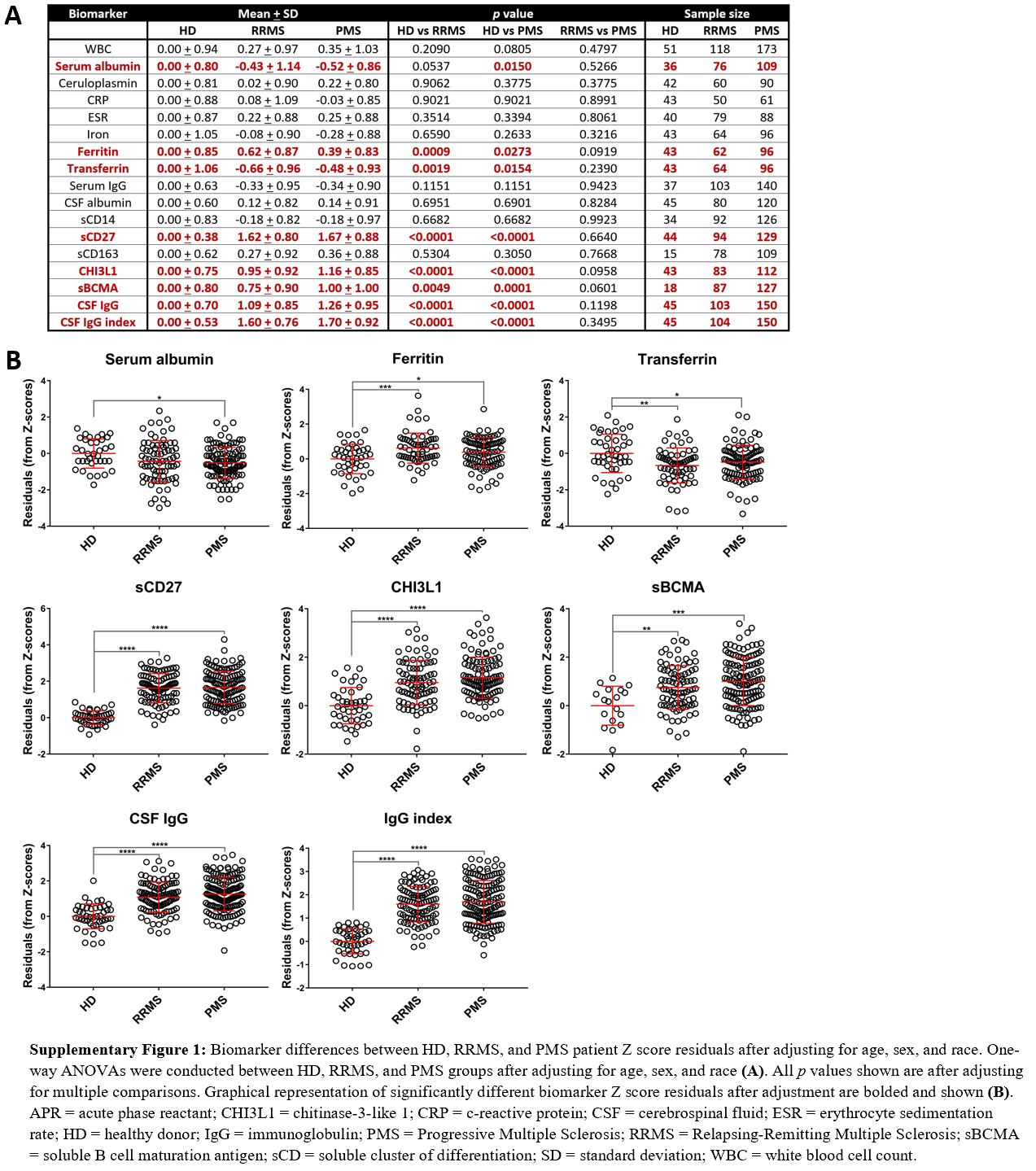

Supplement: Supplementary file 1 [file Image_1.JPEG]

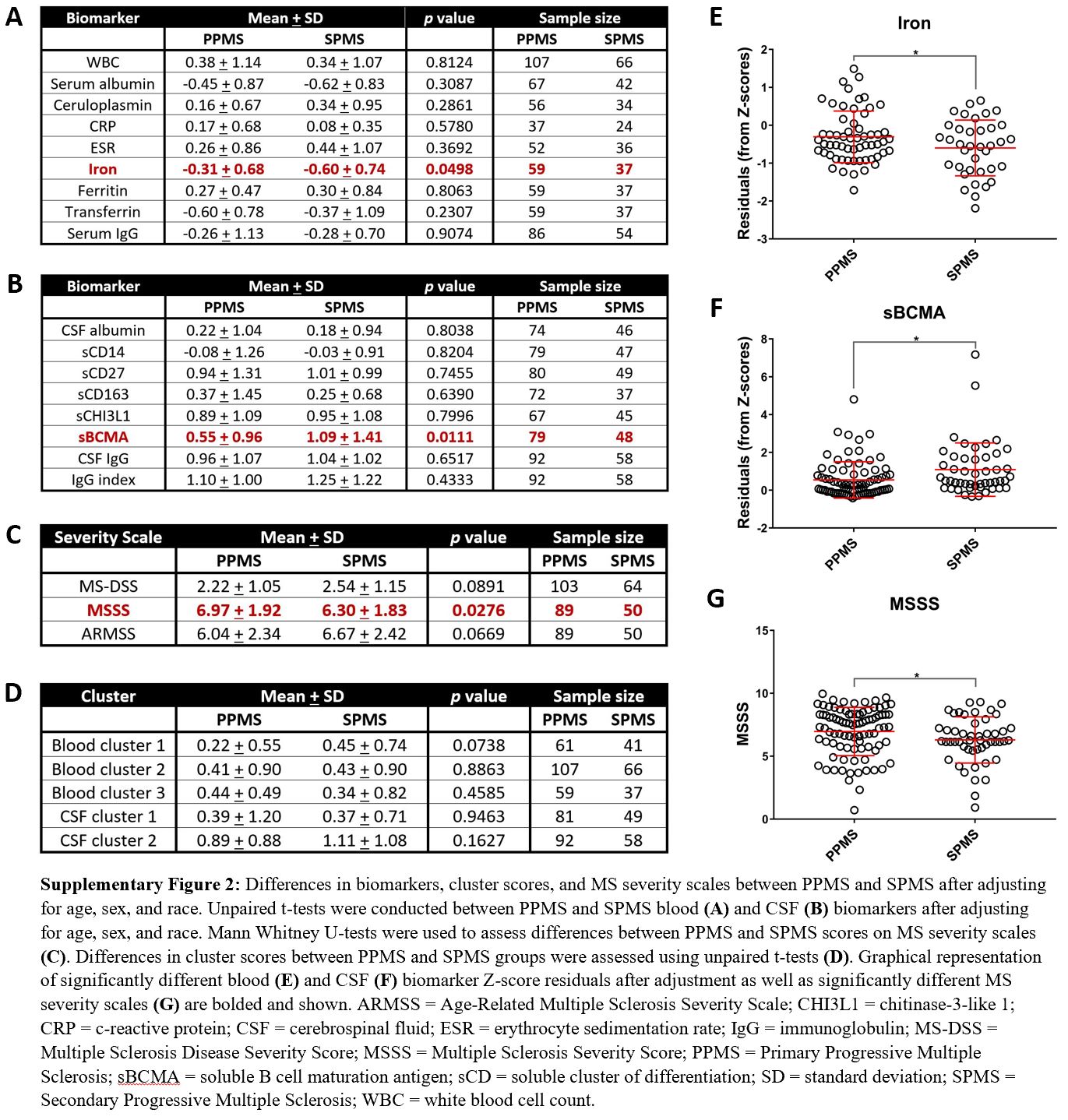

Supplement: Supplementary file 2 [file Image_2.JPEG]

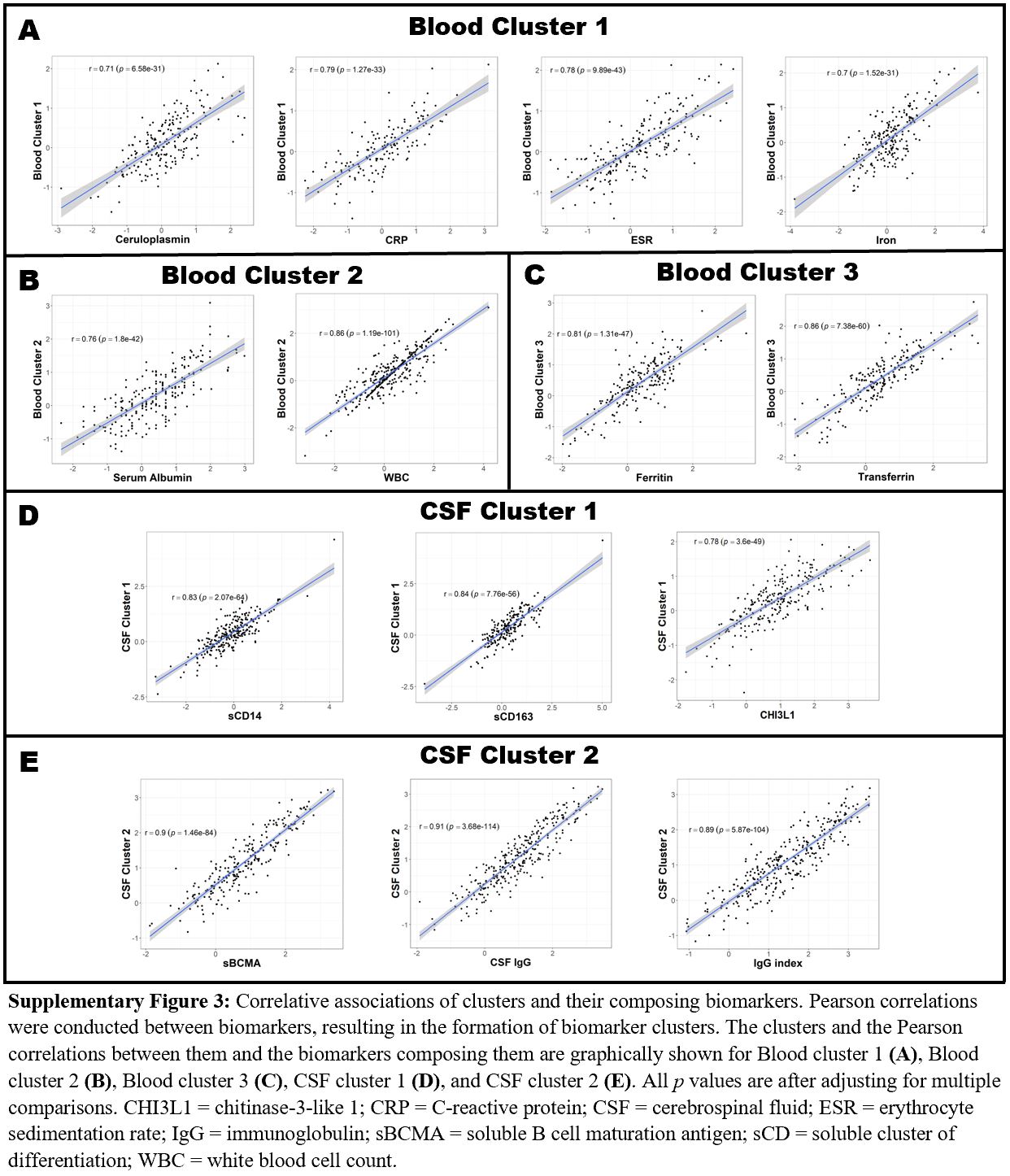

Supplement: Supplementary file 3 [file Image_3.JPEG]

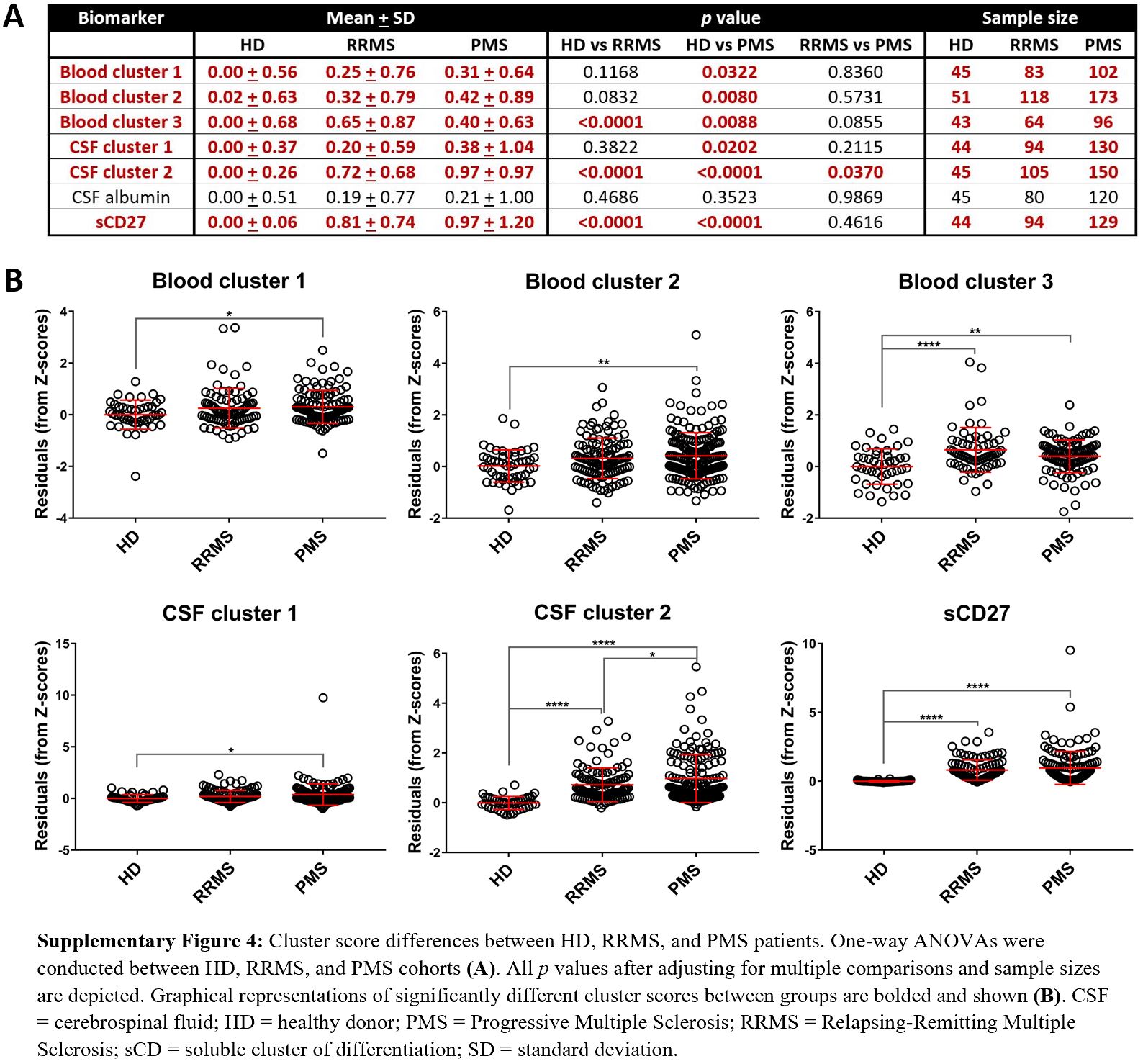

Supplement: Supplementary file 4 [file Image_4.JPEG]

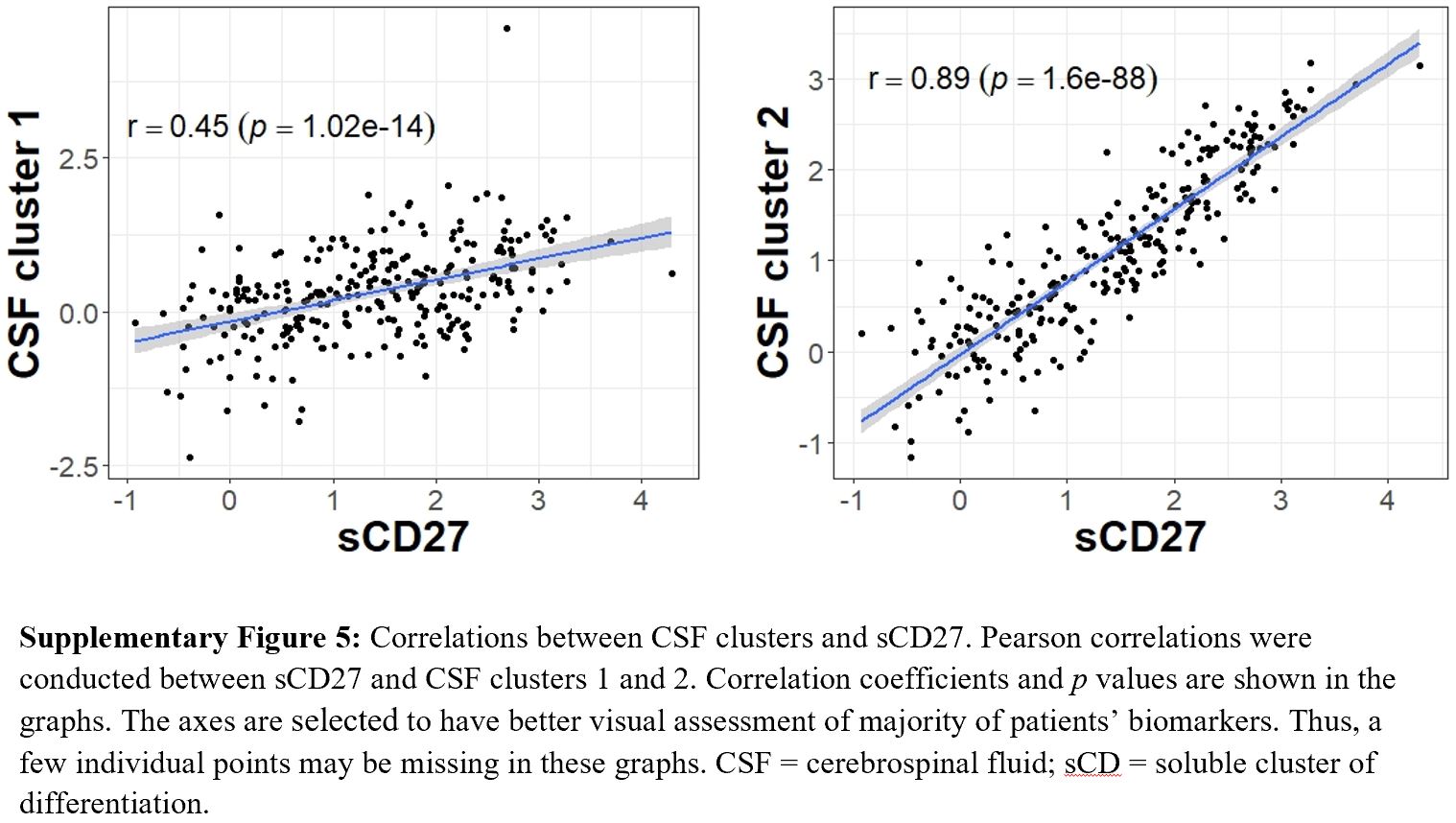

Supplement: Supplementary file 5 [file Image_5.JPEG]
